# Assessing diagnostic value of microRNAs from peripheral blood mononuclear cells and extracellular vesicles in Myalgic Encephalomyelitis/Chronic Fatigue Syndrome

**DOI:** 10.1038/s41598-020-58506-5

**Published:** 2020-02-07

**Authors:** Eloy Almenar-Pérez, Leonor Sarría, Lubov Nathanson, Elisa Oltra

**Affiliations:** 1 Escuela de Doctorado, Universidad Católica de Valencia San Vicente Mártir, Valencia, Spain; 2 Institute for Neuro Immune Medicine, Dr. Kiran C. Patel College of Osteopathic Medicine, Nova Southeastern University, Fort Lauderdale, Florida USA; 3 School of Medicine, Universidad Católica de Valencia San Vicente Mártir, Valencia, Spain

**Keywords:** miRNAs, Diagnostic markers

## Abstract

Myalgic Encephalomyelitis/Chronic Fatigue Syndrome (ME/CFS) is a debilitating multisystemic disease of unknown etiology, affecting thousands of individuals worldwide. Its diagnosis still relies on ruling out medical problems leading to unexplained fatigue due to a complete lack of disease-specific biomarkers. Our group and others have explored the potential value of microRNA profiles (miRNomes) as diagnostic tools for this disease. However, heterogeneity of participants, low numbers, the variety of samples assayed, and other pre-analytical variables, have hampered the identification of  disease-associated miRNomes. In this study, our team has evaluated, for the first time, ME/CFS miRNomes in peripheral blood mononuclear cells (PBMCs) and extracellular vesicles (EVs) from severely ill patients recruited at the monographic UK ME biobank to assess, using standard operating procedures (SOPs), blood fractions with optimal diagnostic power for a rapid translation of a miR-based diagnostic method into the clinic. Our results show that routine creatine kinase (CK) blood values, plasma EVs physical characteristics (including counts, size and zeta-potential), and a limited number of differentially expressed PBMC and EV miRNAs appear significantly associated with severe ME/CFS (p < 0.05). Gene enrichment analysis points to epigenetic and neuroimmune dysregulated pathways, in agreement with previous reports. Population validation by a cost-effective approach limited to these few potentially discriminating variables is granted.

## Introduction

Myalgic Encephalomyelitis/Chronic Fatigue Syndrome (ME/CFS) is a disease characterized by long-lasting (over 6 months) persistent debilitating widespread fatigue, which is exacerbated by exercise (post-exertional malaise or PEM), and not alleviated by rest^1–5^. It has been classified by the International Classification of Diseases, Tenth Revision, Clinical Modification (ICD-10-CM) with code R53.82 or G93.3 if post-viral^6^. It affects at least 0.23 to 0.41% of the population worldwide^7,8^, although numbers can reach up to 5% depending on the diagnostic criteria and geographic locations, according to a recent revision by the EUROpean Myalgic Encephalomyelitis Network (EUROMENE) epidemiology working group^9^. The disease strikes people of all ages and within all socio-economic levels, being more common in women (female:male ratios between 2:1 and 6:1)^7–10^. ME/CFS is considered a life-long disabling disease, since only about 5% of patients return to their previous state of health^11^.

The confusion among General Health Practitioners (GPs) due to multiple clinical definitions^12^ and the large number of comorbidities, such as fibromyalgia, IBS (irritable bowel syndrome), POTS (postural orthostatic tachycardia syndrome), depression, etc, stigmatized the disease, delaying diagnosis and treatment of patients. This highlights the urgent need of unbiased, specific methods to diagnose ME/CFS in a clinical setting.

However, despite a considerable number of studies showing significant differences at the immune and metabolic levels^13–16^, and the report by Institute of Medicine (IOM) of the Academy of Sciences USA stressing that the nature of ME/CFS is a physiologic/medical and not a psychiatric illness^4,5^, current diagnostic methods still rely on ruling out other diseases with similar symptoms.

MicroRNAs, miRNAs or miRs are short non-coding RNAs (20–24 nt-long) with attributed gene expression regulatory functions^17,18^. Their abnormal expression in disease is already quite established^19–21^, making them attractive biomarker candidates in many scenarios, ME/CFS included.

Our group and others have explored the possibility to identify miRNomes or molecular miRNA profiles for ME/CFS and comorbid diseases in the past, by analyzing PBMC (peripheral blood mononuclear cell) contents or by evidencing their presence in body fluids (plasma, serum and cerebrospinal fluid)^22–27^. All body fluids typically contain free miRNAs, miRNAs complexed with proteins, but also miRNAs packed into extracellular vesicles (EVs). The International Society for Extracellular Vesicles or ISEV endorses “extracellular vesicle (EV) as the generic term for particles naturally released from the cell that are delimited by a lipid bilayer and cannot replicate, i.e. do not contain a functional nucleus”^28^. The EV mix is known to contain exosomes, microvesicles, microparticles, ectosomes, oncosomes, apoptotic bodies, and many membranous structures with other names^28^.

The presence of the protective lipid bilayer in EVs constitutes a physical barrier from RNA-degrading enzymes, possibly leading to improved replicability of assays based on RNA quantitation. In addition, some of these vesicles, under the name of exosomes, are known to directionally pack miRNAs and to sustain and spread disease^29–32^, and therefore may provide biomarker advantages for liquid biopsy-based diagnostic methods. As EVs in blood may have their origin in any body cell, including the microbiome and other hosted organisms, their content could potentially inform of dysbiosis in general, in addition to immune dysfunctions^19–21,33^. This last point seems of particular importance in multisystemic diseases, such as ME/CFS, with patients presenting neuro-immune alterations, hormonal imbalances, debilitated muscles, leaky guts, etc. Despite its potential, no study has yet been performed to systematically evaluate particular cargo miRNAs in EVs associating with ME/CFS.

This study aimed to evaluate, for the first time, ME/CFS miRNomes in EVs from severely ill patients and compare/contrast them with those of their PBMCs as a way to assess blood fractions with optimal diagnostic power, for a rapid translation of a miR-based method to diagnose ME/CFS into the clinic. At the same time, the information obtained from EV content analysis could lead to the identification of molecular targets, improving our understanding of the cross-talk between a dysfunctional immune system and the rest of the organism in ME/CFS pathophysiology.

## Results

### Population demographics

This study evaluated blood samples from a cohort of 30 participants (15 severely affected ME/CFS patients and 15 population age-matched healthy subjects), obtained from the monographic UK ME Biobank^34,35^. ME/CFS diagnosis was assessed with Canadian Consensus^2^ and/or CDC-1994 (“Fukuda”) criteria^1^, as detailed in methods. Included individuals were all women with an average age of 46.8 (age range 38–53) and 45.2 (age range 18–52) years, respectively. Median ages were 48 years for the ME/CFS group and 47 for the healthy control group (HC). Average time from disease onset was 17.5 (range 1.5–30.9) years, with a median value of 18.4 years.

### Clinical variables differences

As expected, health survey SF-36^36^ and General Health Questionnaire (GHQ)^37^ scores, including Likert scale^38^ for the GHQ, scores clearly separated ME/CFS and HC groups (p < 0.05) in all fields covered by these questionnaires, indicative of serious compromised health of ME/CFS participants (details are shown on Table 1).

**Table 1:** SF-36 quality of life. GHQ (General Health Questionnaire) and GHQ with Likert scale results for the cohort studied, healthy contros (HC) and ME/CFS patients (N = 15/group).

SF-36 Test	HC		ME/CFS		p value
	Score	SD	Score	SD	
Physical Functioning (PF)	56.8	2.0	21.6	3.0	1.29E-06
Role Physical (RP)	56.9	0.8	21.2	0.0	1.86E-07
Bodily Pain (BP)	56.9	5.0	32.4	12.2	3.69E-05
General Health (GH)	58.9	6.5	26.4	4.1	1.87E-14
Vitality (VT)	58.5	3.9	25.9	5.1	3.13E-06
Social Functioning (SF)	56.7	2.6	19.4	3.8	7.19E-07
Role Emotional (RE)	55.9	0.9	44.0	17.8	3.59E-03
Mental Health (MH)	55.1	2.6	45.4	11.9	1.44E-02
Physical Component Summary (PCS)	57.6	3.7	18.3	5.1	2.58E-08
Mental Health Component Summary (MCS)	55.6	2.2	43.3	12.7	4.06E-05
**GHQ test**	**Score**	**SD**	**Score**	**SD**	**p value**
scale A - Somatic Symptoms	0.3	0.8	3.7	2.9	5.28E-04
scale B - Anxiety and insomnia	0.1	0.3	1.6	2.3	5.22E-03
scale C - Social dysfunction	0.0	0.0	2.9	2.9	7.96E-05
scale D - Severe depression	0.0	0.0	0.8	1.4	1.80E-02
summary	0.3	0.8	9.1	8.1	2.31E-04
**GHQ test with Likert scale**	**Score**	**SD**	**Score**	**SD**	**p value**
scale A - Somatic Symptoms	2.8	1.9	12.5	5.8	1.16E-05
scale B - Anxiety and insomnia	3.1	2.1	6.5	5.4	6.66E-02
scale C - Social dysfunction	6.5	0.3	11.1	5.9	5.61E-03
scale D - Severe depression	0.0	0.3	2.7	4.2	2.10E-02
summary	12.5	3.2	32.8	19.1	3.64E-04

Mean scores, standard deviations (SD) and p values are shown.

Among the 30 clinical parameters evaluated at the UK Biobank in the blood samples studied, only creatine phosphokinase (CPK) levels showed statistically significant differences between groups (p < 0.05), with increased levels by approximately 1.5-fold in ME/CFS patients. Although no other variable of this type showed significant differences between groups, a tendency towards increased eosinophil counts, and lower levels for alkaline phosphatase and free thyroid hormone T4 levels, were noticed in ME/CFS patients. All these three variables presented p values for group differences below 0.1 (Supplementary Table S1). Individual values for these 4 analytical blood parameters were plotted for an improved appreciation of the differences found (Supplementary Fig. S1).

### Plasma EV features differ between ME/CFS and HCs

Since the final goal of the study was to assess the diagnostic value of miRNA profiles from PBMCs and plasma EVs in severe ME/CFS patients and since the miRNA cargo in EVs depends on the isolated vesicle subpopulations^38^, we decided that characterization of the isolated EVs to be used for RNA extraction was necessary.

Based on previous reports showing the superiority of precipitating-based methods *vs* other more laborious classic methods, in terms of EV yields^39^, and our interest for rapidly implementing a technically simple diagnostic method in a clinical context, we decided that the use of a precipitating approach for EV isolation was the most appropriate. Thus, directed by Helwa *et al*. results who compared ultracentrifugation and three different commercial PEG (polyethylene glycol) precipitating methods^40^, in relation to the starting volume of plasma, we chose Invitrogen’s Total Exosome Isolation Reagent (TEIR) and fixed 0.5 mls of plasma as the minimum required volume for our project (see Methods for further details).

**Figure 1:**
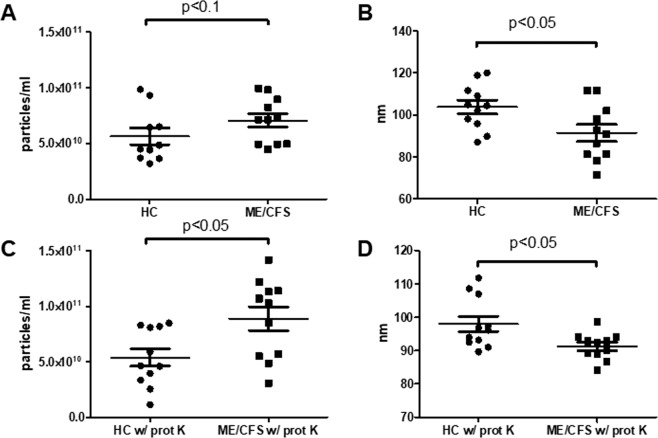
Nanoparticle Tracking Analysis (NTA) of human plasma EVs. Quantitation (**A**,**C**) and size (**B**,**D**) of EVs isolated from either healthy controls (HC) or ME/CFS patients, using a protocol without (**A**,**B**) or with (**C**,**D**) proteinase K treatment (w/prot K). Analysis was performed with a NanoSight NS300, Malvern Panalytical. Means are shown by horizontal bars, whiskers indicate standard deviations (SD).

As shown in Fig. 1A, the EV yields obtained from equivalent volumes of plasma from ME/CFS samples were higher than those isolated from healthy controls (HCs), although not reaching statistical significance (p > 0.05). Interestingly, isolated EVs showed statistically significant size differences, being on average smaller in the ME/CFS group than in the HC’s (Fig. 1B). To rule out that the observed results could derive from differences in plasma protein content interfering with the isolation method, we proceeded to repeat the procedure, this time including a pre-treatment with proteinase K (an optional step recommended by the vendor when using protein-rich fluids). By introducing this optional treatment, we could confirm that qualitative differences exist, both, in number of vesicles and size, in plasma of ME/CFS patients with respect to HCs (p < 0.05) (Fig. 1C,D).

**Figure 2:**
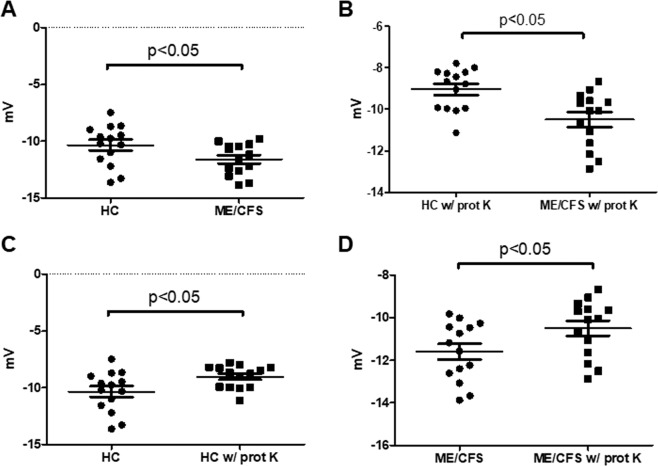
Zeta potential analysis of human plasma EVs. EVs were either isolated in the absence of proteinase K (**A**) or including a proteinase K pre-treatment (w/ prot K) (**B**), as labeled. Study groups included healthy controls (HC) or ME/CFS patients, as indicated. Means are shown by horizontal bars, whiskers indicate standard deviations (SD).

To further characterize the seemly diverse isolated EVs, we measured their zeta potential, a parameter reflecting the surface charge of the vesicle which may be informative of membrane composition differences and indicative of vesicle stability or the likelihood of these colloidal suspensions to aggregate. Although the values obtained fit, as an overall, those reported in the literature for zeta potential of EVs isolated from human plasma (−10 to −50 mV)^41^, significant differences were found between values of either group studied. In particular, ME/CFS’s EVs presented more negative values than the EVs from the HC group, both, if they were isolated in the absence (Fig. 2A) or presence (Fig. 2B) of proteinase K. This suggests that differences in the lipid and/or other membrane components exist and that, as an overall, ME/CFS EVs are possibly slightly more stable than EVs from HCs. Furthermore, when the EVs from each group were independently compared, in reference to pretreatment with proteinase K or in its absence, significant differences were observed for both the HC (Fig. 2C) and the ME/CFS’s group (Fig. 2D). Since in both cases the proteinase K pretreatment led to more positive results (less negative zeta values) the results are interpreted as evidence for the role of protein factors present in plasma EVs determining membrane electronegativity, stability in solution and, possibly, their function.

### Isolated plasma EVs showed exosome features

**Figure 3:**
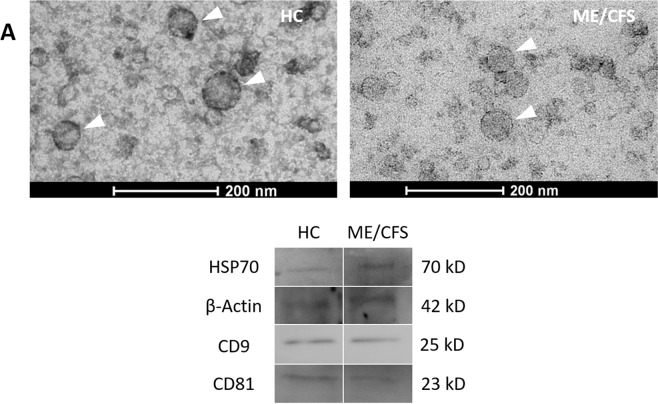
Characterization of human plasma EVs from healthy controls (HC) or ME/CFS patients. TEM (Transmission Electron Microscopy) (**A**) (left HC, right ME/CFS) and western blot analysis with anti-CD9 (Cell Guidance Systems, cat.EX201), anti-CD81 (Santa Cruz Biotechnology, cat. sc-23962), anti-HSP70 antibody (Santa Cruz Biotechnology, cat. sc-7298) or anti-β-Actin antibody (Santa Cruz Biotechnology, cat. sc-130657) (**B**), as primary antibodies (1:200). EVs are indicated by white triangles.

Isolated EVs TEM (Transmission Electron Microscope) images and Western-blot analysis show that the isolated EV fractions, from both ME/CFS and HC plasmas, present several exosome features, including morphology (bilayered membrane “saucer shape” vesicles), size (diameter 30–100 nm), and presence of the tetraspanins CD9 and CD81 (Fig. 3). This vesicle subset mediating long-range communication, reportedly carrying differential miRNA cargos in disease^30–33^, have been shown to have potential diagnostic value in other contexts and, therefore, were of interest to our project. However, as our final goal was to find a technically simple diagnostic strategy with independence of the isolated EV subsets by our method of choice, no further efforts in exosomal marker detection were invested, nor additional purification steps were performed.

### RNA samples in miRNA profiling assays

RNA yields obtained from the 30 PBMC samples ranged between 810–2800 ng, and those from EVs varied between 102–514 ng. Only PBMC RNA samples with RIN (RNA integrity number)  values above seven were considered for further analysis. The criterion was fulfilled by 28 out of 30 samples, indicating an overall acceptable quality of the PBMC samples provided by the UK ME Biobank. Both samples showing low RIN numbers belonged to the HC group. Therefore, the final number of RNA samples from PBMCs considered suitable for downstream analysis was of 28 (15 ME/CFS and 13 HCs) (Supplementary Table S2).

Since no quality control can be applied to total RNA obtained from EVs, and their small RNA profiles presented similar characteristics, independently of the protocol used for EV isolation (with or without proteinase K pre-treatment), or the group analyzed (ME/CFS or HC) (Supplementary Fig. S2), all 30 RNA samples from EVs (15 ME/CFS and 15 HCs) were included in the microRNA profiling analysis.

### Differences in RNA pools between PBMCs and plasma EVs

Total small RNA content in EVs showed similar patterns regardless of the group (ME/CFS or HCs) (Supplementary Fig. S2). No major species (size-wise peaks) were detected on the range of 4 to >150 nts. By contrast, a broad peak in the 20–40 nt and a second narrow peak around 60 nts were observed in total RNA prepared from PBMCs (Supplementary Fig. S3) supporting differential small RNA sorting into vesicles as formerly depicted by other authors^29^.

Upon applying recommended normalization parameters (see methods for details), only 136 miRNAs from PBMC’s and 639 out of 798 from EV’s were used for differential expression analysis (Supplementary Dataset 1). Next, we removed samples showing counts below background in 40% or more of the normalized miRNAs. The loses were three (two ME/CFS samples and one from the HC group) among PBMCs and another three in EVs (coincidentally also two ME/CFS samples and one from the HC group). This new filter reduced the total number of analyzed samples to 13 ME/CFS and 12 HCs in PBMCs, and to 13 ME/CFS and 14 HCs in EVs. Samples eliminated from analysis did not correspond to the same donors in PBMC and EV fractions indicating that the quality of the original biobanked samples was not related to this finding.

**Figure 4:**
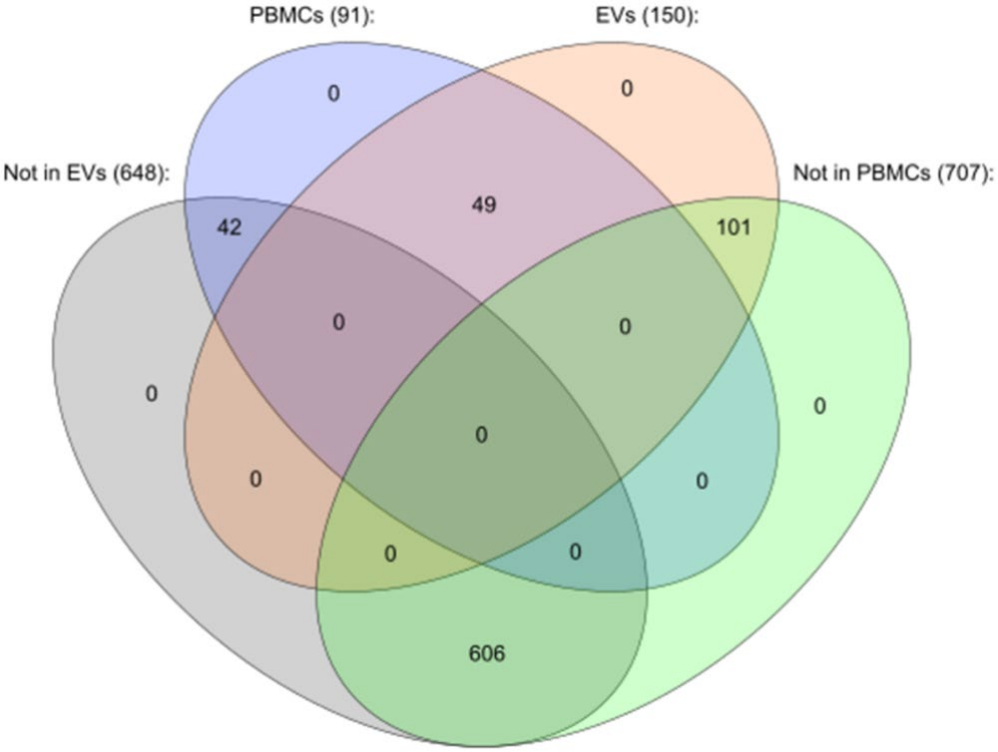
Venn diagram showing miRNAs found in PBMCs and EVs by nanostring analysis. Numbers of miRNAs expressed in either fraction after applying strict normalization filters are indicated. Drawn with Cytoscape version 3.6.0 and the Venn & Euler diagram package, version 1.0.3.

A third filter was applied to strictly include miRNAs presenting readings in at least 75% of samples of either group (ME/CFS or HCs), finding only 91 miRNAs in PBMCs and 150 in EVs meeting these criteria. 49 of them were present in both sets of samples, while 42 and 101 were exclusive of PBMCs or EVs, respectively (Fig. 4) (Supplementary Dataset 2). These miRNAs were considered to be robustly and reproducibly expressed in PBMCs and EVs. The restrictive filters applied lead to classify 606 miRNAs out of the 798 tested as not being highly expressed in either PBMCs or EVs, as shown in Fig. 4.

### ME/CFS PBMC miRNomes

Next, we subjected all these 241 miRNAs to differential expression (DE) analysis (as described in Methods). Since the reduced number of samples could lead to discard miRNAs, which although not reaching statistical significance in t-student or Mann-Whitney tests, might be of relevance to the disease, we proceeded to detect and filter potential outlier values among DE miRNAs (p < 0.1) by applying Chauvenet’s criterion^42^. This method consists of detecting an acceptable band of data around the mean. Values falling outside that band (>2-fold SD absolute values) are considered outliers.

**Figure 5:**
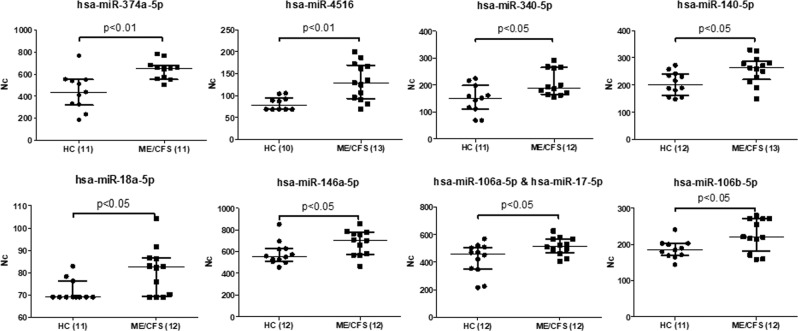
Overexpressed miRNAs in PBMCs of ME/CFS with respect to HCs, after adjustment of outlier values according to Chauvenet criterion, (Mann Whitney, p < 0.05). The number of samples analyzed per group for either miRNA appears between brackets. Medians are shown by horizontal bars, whiskers indicate interquartile ranges (IQR).

**Figure 6:**
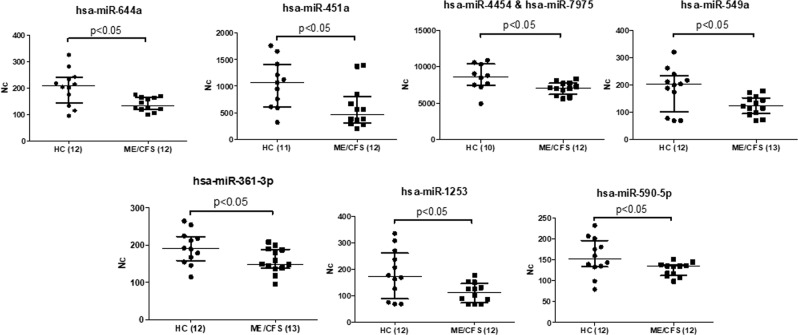
Under expressed miRNAs in PBMCs of ME/CFS with respect to HCs, after adjustment of outlier values according to Chauvenet criterion, (Mann Whitney, p < 0.05). The number of samples analyzed per group for either miRNA appears between brackets. Medians are shown by horizontal bars, whiskers indicate interquartile ranges (IQR).

Statistical analysis of the DE miRNAs in PBMCs, after this adjustment, showed 17 miRNAs presenting significant differences (p < 0.05), 9 of them being overexpressed (Fig. 5 & Supplementary Table S3); and 8 under expressed (Fig. 6 & Supplementary Table S4) in the ME/CFS group. The miRNAs showing significantly increased values (p < 0.05) were: hsa-miR-374a-5p, hsa-miRNA-4516, hsa-miR-340–5p, hsa-miR-140-5p, hsa-miR-18a-5p, hsa-miR-146a-5p, hsa-miR-106a-5p & hsa-miR-17-5p and hsa-miR-106b, by decreasing order of significance. Differences varied between 20 and 65% (Supplementary Table S3). While those miRNAs showing significantly decreased values were: hsa-miR-644a, hsa-miR-451a, hsa-miR-4454 & hsa-miR-7975, hsa-miR-549a, hsa-miR-361-3p, hsa-miR-1253 and hsa-miR-590-5p, also cited by strict order of decreasing significance. Differences of expression in this last group varied between 13 and over 200%. In addition, hsa-miR-21-5p presented a p-value > 0.05 and <0.1, showing a tendency to be overexpressed in ME/CFS PBMCs with respect to those of HCs (Supplementary Table S3).

Interestingly enough, three of the identified DE miRNAs (hsa-miRNA-146a-5p, hsa-miR-17-5p and hsa-miRNA-451a) had been reported dysregulated by other authors in both, ME/CFS or fibromyalgia (FM) patients^22,23,43^.

### ME/CFS plasma EV miRNomes

**Figure 7:**
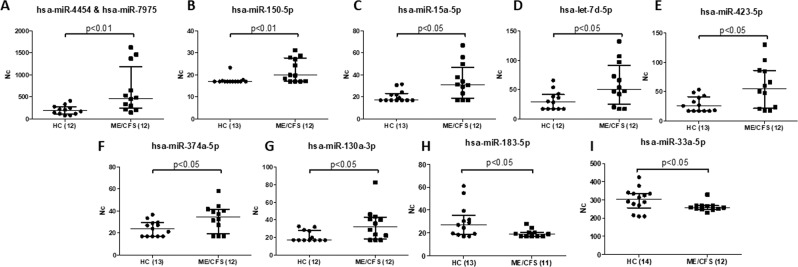
DE miRNAs in plasma EVs of ME/CFS and HCs, after adjustment of outlier values according to Chauvenet criterion, (Mann Whitney, p < 0.05). Overexpressed (A–G), and under expressed (H,I) miRNAs are shown. The number of samples analyzed per group for either miRNA appears between brackets. Medians are shown by horizontal bars, whiskers indicate interquartile ranges (IQR).

Similarly, we found ten DE (p < 0.05) miRNAs in EVs: eight overexpressed (Fig. 7A through 7G, and two under expressed, (Fig. 7H,I), in ME/CFS. The miRNAs showing significantly increased values (p < 0.05) were: hsa-miR-4454 & hsa-miR-7975, hsa-miR-150-5p; hsa-miR-15a-5p, hsa-let-7d-5p, hsa-miR-423-5p, hsa-miR-374a-5p and hsa-miR-130a-3p. The differences in expression found for this group varied between 17 and 243%. (Supplementary Table S4). While the two miRNAs showing significant under expressed values in ME/CFS EVs (p < 0.05) were: hsa-miR-183-3p and hsa-miR-33a-5p. Calculated under expressed values were 47 and 17%, respectively (Supplementary Table S4). Large differences in certain miRNA levels were noticed between patients (Fig. 7A,C–E,G), which may indicate the existence of patient subgroups.

In addition, hsa-miR-21-5p, hsa-miR-320e, hsa-miR-203a-5p, hsa-miR-185-5p, hsa-miR-607, hsa-let-7g-5p, hsa-miR-126-3p, hsa-miR-223-3p, hsa-miR-369-3p and hsa-miR-93-5p all presented p-values > 0.05 and <0.1 (listed by decreasing level of significance). Hsa-miR-21-5p, hsa-miR-320e, hsa-miR-185-5p, hsa-let-7g-5p, hsa-miR-126-3p, hsa-miR-223-3p, and hsa-miR-93-5p were overexpressed, while hsa-miR-203a-5p, hsa-miR-607 and hsa-miR-369-3p were under expressed in ME/CFS EVs with respect to those of HCs (Supplementary Table S4).

Three of the significant DE miRNAs in PBMCs were also DE in EVs (hsa-miR-374a, hsa-miR-4454 & hsa-miR-7975, bolded in Supplementary Tables S3 and S4) and one among those showing a tendency (p < 0.1): hsa-miRNA-21-5p, also did. Among these miRNAs dysregulated, both in PBMCs and EVs, hsa-miR-374a and hsa-miRNA-21-5p presented overexpression in both fractions, while hsa-miR-4454 & hsa-miR-7975 appeared under expressed in PBMCs and overexpressed in EVs (Supplementary Tables S3 and S4). At present, the significance of these differences is not understood.

Supplementary Fig. S4 shows the heatmaps of miRNAs presenting DE between ME/CFS and HCs (p value < 0.05) either in PBMCs or EVs to illustrate the described differences in miRNA levels.

A minimum of 10 samples per group were included for all miRNAs evaluated in PBMCs, while a minimum of 11 were included in EVs at all times.

### Potential pathways and molecular functions targeted by DE miRNAs

In an effort to determine the physiological significance of the identified DE miRNAs in ME/CFS, we performed Gene Ontology (GO) enrichment analysis using the genes potentially targeted by the DE miRNAs with p values < 0.05, using the miRDIP and Reactome pathway databases, as detailed in Methods.

Top pathway hits are shown in Supplementary Datasets 3 through 6. Interestingly, main affected pathways in PBMCs include post-transcriptional silencing by small RNAs, circadian clock, chromatin organization and chromatin modifying enzymes, in addition to several pathways related to the X-chromosome encoded *MECP2* (methyl-CpG binding protein 2) gene, both, among miRNAs over and under expressed in ME/CFS. The fact that *MECP2* is subject of X-chromosome inactivation is supportive of potential environmental sex-biased effectors for this acquired disease.

In addition, we also found *MECP2* pathways among top hits in DE miRNAs overexpressed in EVs with p < 0.05. The finding that neuronal and endocrine system pathways are among DE miRNA hits in ME/CFS EVs, might be indicative of defects in endocrine tissue functioning, as many patients report. Neurotrophic receptor tyrosine kinase (*NTRK*) signaling seems to be also impacted by significant DE miRNAs (p < 0.05) both in PBMCs and EVs which could relate to patient symptoms such as cognitive disability and sensorial dysfunctions (altered pain sensitivity)^44^.

### Diagnostic value assessment of evaluated variables

Since the main goal of the study was to determine the potential diagnostic value of the variables measured, we conducted response operating characteristic (ROC) analyses for each of the miRNAs showing significant DE between ME/CFS and HC individuals (p < 0.05), at individual level (miRNAs shown in Fig. 5 through 7 and Supplementary Tables S3 and S4).

**Figure 8:**
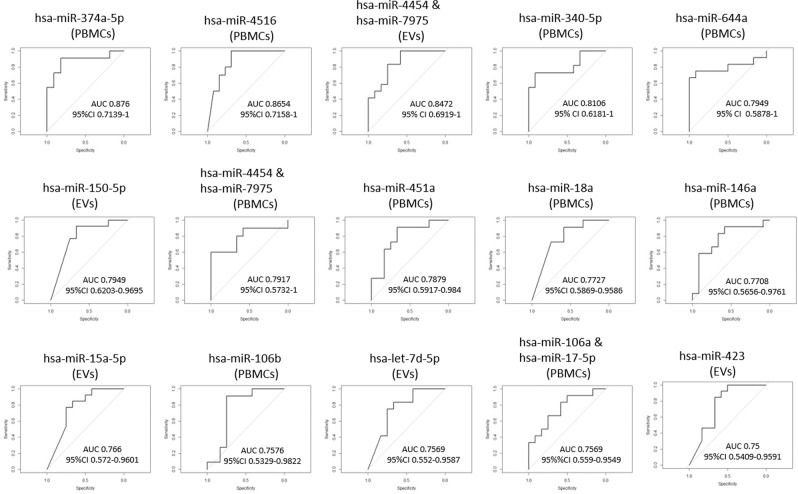
Calculated area under the curve (AUC) for individual DE microRNAs (p < 0.05). AUCs > 0.75 and 95% confidence interval (CI) values are shown. Blood fractions presenting differentially expressed miRNAs (PBMCs or EVs) are indicated between brackets.

As shown in Fig. 8, fifteen of the twenty-seven significant DE miRNAs detected in this study present values for the area under the curve (AUC) above 0.75 indicating that they have an acceptable capacity to discriminate blood samples of ME/CFS patients and HCs with respect to their miRNA content. The microRNAs that present improved discrimination capacity are: hsa-miR-374a-5p (AUC: 0.876, 95% C.I:0.7139–1), hsa-miR-4516 (AUC: 0.8654, 95% C.I: 0.7158–1), hsa-miR-4454 & hsa-miR-7975 (AUC: 0.8472, 95% C.I: 0.6919–1) and hsa-miR-340 (AUC: 0.8106, 95% C.I 0.6181–1), all belonging to the PBMCs fraction, with the only exception of hsa-miR-4454 & hsa-miR-7975 which could be considered the potential top biomarker candidate in the EV fraction, with AUC above 0.8.

### Validation of DE miRNAs predicted target genes

**Figure 9:**
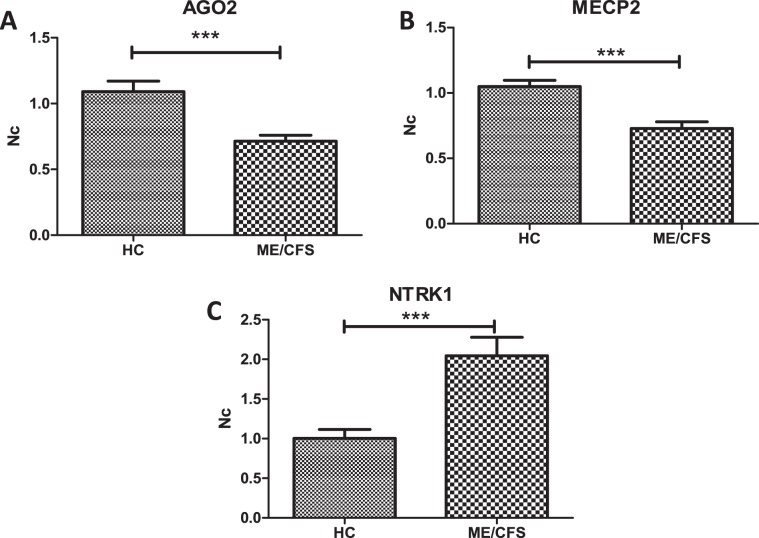
Quantitation of mRNA levels in PBMCs of ME/CFS with respect to HCs. RT-qPCR amplification of selected genes in top target pathways for DE PBMC miRNAs is shown. Expression levels were normalized to endogenous GAPDH levels, as described in Methods. Means and standard deviations (SD) are shown (N = 10/group, t-test, p < 0.001).

In order to determine the impact of DE miRNAs on PBMCs transcriptome, we designed sets of primers to quantitate the levels of representative genes within commonly affected pathways across DE miRNAs hits (Supplementary Datasets 3 and 4), by RT-qPCR. In particular we chose: the *AGO2* gene from the silencing by small RNAs category, including R-HSA-426496 and R-HSA-426486 Reactome pathways; the *MECP2* gene from MECP2 related pathways, including R-HSA-9022707, R-HSA-9022699, R-HSA-9022692, R-HSA-8986944, R-HSA-9022534, R-HSA-9005891, R-HSA-9022538, R-HSA-9022927, R-HSA-9022537 and R-HSA-9022702 Reactome pathways; and the *NTRK1* gene from R-HSA-187037 and R-HSA-9028731 neurotrophin signaling Reactome pathways. Figure 9 shows that *AGO2* and *MECP2* are under expressed, while *NTRK1* levels are increased in ME/CFS PBMCs. As discussed above, the connection of these pathways with patient symptoms suggests their contribution in the pathophysiology of ME/CFS.

## Discussion

Researchers and clinicians seem to agree on the urgent need for unbiased, specific diagnostic biomarkers for ME/CFS to expedite patient diagnosis and treatment, and to abolish disease stigmas for good^45^. There is also a consensus on the advantages that miRNA profiles from different blood fractions, EVs included, exhibit as molecular diagnostic candidates, and yet, a paucity of studies focusing on ME/CFS miRNA profiling is detected^22,24–27^.

A limitation for biomarker discovery studies in ME/CFS is that they have so far consisted of pilot studies with low number of participants, and the present work, with an N of 30 participants, is no exception. The importance of our design, however, relies in that, for the first time, miRNomes in PBMCs and EVs were studied in the same exact individuals, in addition to 34 blood analytical variables and 6 additional EV features (yields, size and zeta values, either in presence or absence of proteinase K treatment), and several health questionnaires (SF-36 & GHQ28), leading to the most complete phenotype registry of severely affected ME/CFS individuals we are aware of.

To prevent additional limitations hampering biomarker discovery in ME/CFS, we tried to minimize patient heterogeneity and biases associated to pre-analytical variables by only including samples from the UK monographic ME biobank, which uses procedures complying with the NINDS (National Institute of Neurological Disorders and Stroke) Common Data Elements (CDEs) for the study of ME/CFS^46^. Diagnosis criteria and standard operating procedures (SOPs) used at this facility have been briefly summarized in the methods section. For further details, readers are referred to publications from the biobank^34,35^.

Blood creatine phosphokinase (CK) levels appeared significantly reduced in our ME/CFS studied cohort (t-student, p < 0.05, Supplementary Table S1), in agreement with the recent report by Nacul *et al*. including evaluation of this variable in 56 severe ME/CFS patients^47^. Serum CK could therefore constitute a biomarker for severe ME/CFS cases (96% sensitivity) albeit with low specificity (<50%), as these authors report. Although a bias coming from patient inactivity cannot be ruled out at this point, the fact that a multivariate model controlling for activity maintains its significance, in addition to its potential to explain PEM in ME/CFS warrants further exploration^47^.

Because technical simplicity is desirable for clinical tests, we decided that rather than pursuing the isolation of highly purified EV subpopulations obtained through lengthy protocols, the use of optimized commercial kits rendering high EV yields by simple-fast procedures was preferable.

It must be noted that availability of these highly valuable samples is quite restricted. This was an additional reason for choosing TEIR reagent as the EV isolation method, as it provides highest yields from only 0.5 mls of plasma when compared to other commercial products, or alternative isolation methods^40^. Therefore total amounts of sample could be minimized. Additional authors concur with Helwa *et al*. that EVs isolated with polyethylene glycol (PEG) 6000, as is the case of the commercial product TEIR, render particles of similar size to alternative methods, including ultracentrifugation, ultrafiltration, and gel chromatography, with increased yields, as summarized by Konoshenko *et al*.^48^.

The present study intends to pave the way in exploring blood EV features in ME/CFS, thus, opening the possibility of evaluating miRNA networks affected by ME/CFS pathophysiology in the entire organism. Another main aspect of the project was to determine whether EVs constitute a more sensitive, or otherwise improved fraction than PBMCs, in detecting miRNA changes in ME/CFS, for diagnostic purposes. ROC analysis, however, points towards superior diagnostic value of miRNomes from PBMCs compared to EV’s (Fig. 8). The high variability and low miRNA copy number in EVs with respect to PBMCs (counts threshold of 17 versus 69, Supplementary Dataset 1, PBMC & EV tabs) may, at least partly explain this finding. Surprisingly, yields, size and zeta power values of EVs appeared as disease-discriminating parameters by the same approach (Figs. 1 & 2).

In line with findings in other pathologies, including cancer and neurodegenerative diseases^49,50^, we found higher numbers of EVs in the disease state (Fig. 1A,C). Interestingly enough, a recent pilot study evaluating number and size of EVs in serum also found increased numbers in ME/CFS patients^51^. In addition to increased EV yields, we also observed that the mean size of ME/CFS EVs is reduced (Fig. 1B,D), showing an average diameter below 100 nm. This is also in agreement with the mentioned findings by Castro-Marrero *et al*.^51^. Although we used plasma instead of serum, the parameter differences found seem to coincide, evidencing reproducibility in more than one type of sample and across different cohorts. The question on whether one cell produces EVs of different sizes or does size difference reflect EV production by different cells remains presently unknown.

EV size depends not only on the type of membrane phospholipids but also on the presence or absence of particular membrane proteins^52^ and the difference may have functional consequences. For example, it has been proposed that cytokines found on the EV surface may serve as bar code molecules being recognized by abundant cell-specific cytokine receptors^52,53^. Mass spectrometry (MS) should definitively be included in follow up studies to evaluate potential qualitative differences associating to ME/CFS.

Since the freezing/thawing process may affect the properties of the vesicles, vesicle flocculation included^54^, our experiments were all based on a single thaw step. However, we cannot rule out at present vesicle collapsing in HC as an explanation to their increased size. In fact, EVs from patients presented higher stability (within an incipient instability range) according to their higher zeta potential absolute values (Fig. 2).

The observation that ME/CFS EV zeta values are more negative than those of the HC counterpart suggests that the outer layer phospholipids, or the membrane proteins, or both, contain higher negative charge. Sialic acid on membrane-bound oligosaccharides lead to increased net negative charge of secreted vesicles from tumor cells^54^. It will be interesting to determine if there is an enrichment of sialic acid in ME/CFS EVs.

The fact that pretreatment with proteinase K reduces membrane electronegativity in both ME/CFS and HC points to a contribution of proteins in the net vesicle charge. However, it must be noted that the overall charge in ME/CFS EVs stays higher also upon proteinase K treatment in comparison to treated EVs from HCs (Fig. 2C,D), leaving lipids and carbohydrates as main candidates to explain the observed charge differences between ME/CFS and HCs. Unbiased distribution of lipids in vesicle leaflets and/or differences in sugar ratios are interesting possibilities worth exploring as they could be indicative of different subpopulations in the disease *vs* the healthy state.

Despite the fact that EVs isolated with TEIR reagent include vesicles with exosomal morphology and size-range under transmission electronic microscopy (Fig. 3A), and the presence of the exosomal markers CD9 and CD81 were evidenced by western blotting (Fig. 3B), our procedures were limited at estimating the relative abundance of exosomes and other vesicle subgroups in general, and therefore any potential attribute of exosomes or other vesicle subtype in ME/CFS would be merely speculative.

The total final number of miRNAs that were expressed above background seemed strikingly low, with only 91 in PBMCs and 150 in EVs (Fig. 4), out of the approximately 800 included in the nCounter Human miRNA Panel v3 (Nanostring Technologies, GXA-MIR3-12). As the samples had passed demanding quality controls (Supplementary Table S2), the low number of hits in PBMCs must be due to restricted blood cell-type expression, as described by other authors^55^, together with the strict selective criteria imposed in this study (Supplementary Figs. S3 and S4). Of the 27 miRNAs DE in ME/CFS (p < 0.05) (17 in PBMCs and 10 in EVs), we find, following a database search formerly described by our group^56^, that hsa-miR-18a-5p can be upregulated by the anti-psychotic drug desipramine, while hsa-miR-146a-5p and hsa-miR-150-5p expression can be induced by morphine^56^, raising the possibility that the observed overexpression of these miRNAs derives from patient drug exposure and not from the disease itself. This highlights the enormous importance of detailed medication registry when studying molecular changes in these patients, as recommended by the ME/CFS NINDS CDEs^46^.

The fact that the number of miRNAs detected above background levels found in EVs is larger than in PBMCs was somewhat expected since EV varied cell-source should lead to higher miRNA complexity^39^.

Although due to selective packaging of miRNAs, EV miRNomes could be useful to trace the cells of origin^29,52^, we still need to first understand the “rules” for this directional process. By contrast, some data suggests non-selective miRNA packaging into EVs^57^. Interestingly Ridder *et al*., show, by using an elegant Cre-LoxP-based genetic tracing system, that it is not only the cell-type, but the health status of the individual which may determine molecule directional packaging and function of EVs. In their experiments, the authors evidenced that the transfer of signaling RNA between immune cells and the brain, gut and additional tissues is promoted by inflammatory injuries^58^.

MiRNA tissue-specific expression could provide hints regarding cell origin of plasma EVs. In this regard, Ludwig *et al*., determined the relative abundance of 1997 miRNAs in 61 tissue biopsies of different organs collected post-mortem from two individuals^59^. Based on a tissue specificity index (TSI), the authors found that the majority of miRNAs (82.9%) fell in a middle TSI range, meaning they were neither specific for single tissues (TSI > 0.85) nor housekeeping miRNAs (TSI < 0.5). Many different miRNAs and miRNA families’ expression, however, were predominant in certain tissues. Moreover, miRNAs TSI values of human tissues were well correlated with those of rats, supporting interspecies-conserved tissue-enriched miRNAs^59^. Only five miRNAs resulted highly specific for hematopoietic cells: miR-142, miR-144, miR-150, miR-155, and miR-223. Among them only miRNA-150 appeared DE (p < 0.05) in our EV profiling and miR-223 presented a DE tendency (0.05 > p < 0.1). Using their web server (https://ccb-web.cs.uni-saarland.de/tissueatlas) as a search tool^58^ we found that EV DE miRNAs (p < 0.05): hsa-miR-150-5p, hsa-miR-15a-5p, hsa-miR-183-5p, hsa-miR33a-5p, let-7d-5p, hsa-miR-423-5p, hsa-miR-374a-5p and hsa-miR-130a-3p were predominantly expressed in muscle and fascia, brain and nerves and hormonal glands (mostly thyroid), after quantile normalization. All of them correspond to tissues affected in ME/CFS.

Although hsa-miR-4454 and hsa-miR-7875 are not found in the mentioned database, Polytarchou *et al*., reported detecting hsa-miR-4454 in serum, as a potential marker of ulcerative colitis with higher sensitivity and specificity values than C-reactive protein^60^. This fits with the fact that inflammation frequently associates with ME/CFS^61^. Perhaps, the large differences observed in patient EVs for this miRNA (Fig. 7A) relate to intestinal dysbiosis in some patients. Detailed clinical history registry of participants will be key to answer this question, as pointed by the ME/CFS CDEs^46^.

Only four miRNAs were found in both fractions (PBMCs and EVs). Two of them (hsa-miR-21-5p and hsa-miR-374a-5p) followed the same trend, being overexpressed in both fractions, while the closely related hsa-miR-4454 and hsa-miR-7975 appeared under expressed in PBMCs and overexpressed in EVs which could be explained by directional packaging of immune cells. It seems particularly interesting that hsa-miR-4454 and hsa-miR-7975, linked to inflammation by some authors^60^, appears among the top DE miRNAs with potential diagnostic value according to ROC analysis (Fig. 8). At present, however, we can only speculate on the potential significance of these findings. GO pathway analysis and RT-qPCR results (Fig. 9) points to potential epigenetic and signal transduction mechanisms associating with ME/CFS, in good agreement with the differential methylation patterns formerly detected in ME/CFS patients by our group^62^.

**Figure 10:**
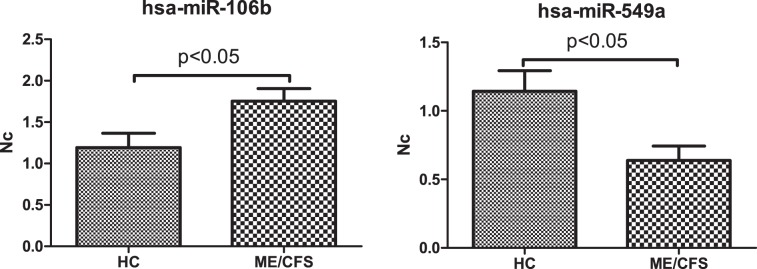
RT-qPCR validation of miRNAs over (left) or underexpressed (right) in PBMCs from ME/CFS participants, according to Nanostring analysis. Means and standard deviations (SD) are shown (N = 10/group, t-test, p < 0.05).

Finally, although, opposite to microarrays, nanostring technology does not require validation of the results obtained by alternate methods^63^, we proceeded, anyway, to test whether differential expression profiles detected by nanostring in our samples, could be assessed by the cost-effective RT-qPCR method. As shown in Fig. 10, both miRNAs randomly picked from the overexpressed (hsa-miR-106b) and the under expressed (hsa-miR-549a) groups were confirmed to be statistically DE in ME/CFS PBMCs. This opens the exciting possibility for a directional low-cost screening method, including only a few miRNAs together with routine blood analytical parameters and some EV features, to evaluate larger ME/CFS cohorts towards population validation of the potential biomarkers of ME/CFS detected here. Inclusion of diseased controls, such as multiple sclerosis (MS), systemic lupus erythematosus (SLE) or other diseases presenting overlapping symptoms will be of relevance in pursuance of ME/CFS specific biomarkers.

## Methods

### Samples & associated clinical data

Ethical approval of the study was granted by two independent Research Ethics Committees: the Public Health Research Ethics Committee DGSP-CSISP, Valencia (Spain), study number UCV_201701 and by the UCL Biobank Ethical Review Committee-Royal Free London NHS Foundation Trust (B-ERC-RF), study number EC2017.01 before the samples were released by the UK ME Biobank. All methods were performed in accordance with relevant guidelines and regulations.

All subjects signed an informed consent before samples could be included in the UK ME Biobank^34,35^.

The samples included in the study consisted of PBMCs (>10^6^ cells) and plasma from 15 severely ill female ME/CFS patients and 15 female, age-population matched healthy subjects, obtained in EDTA blood-collection tubes, by the monographic UK ME Biobank professionals. A total of 90 aliquots: one vial of PBMCs/participant (total of 30 units), and two sets of 1 ml plasma vials/participant (total of 60 aliquots).

As described in the literature, patient recruitment and clinical assessment was mainly performed through the UK National Health Service (NHS) primary and secondary health care services. Compliance with the Canadian Consensus^2^ and/or CDC-1994 (“Fukuda”) criteria^1^ were ensured for patient recruitment. Participants exclusion criteria were as follows: (i) taken antiviral medication or drugs known to alter immune function in the preceding 3 months (ii) had any vaccinations in the preceding 3 months; (iii) had a history of acute and chronic infectious diseases such as hepatitis B and C, tuberculosis, HIV (but not herpes virus or other retrovirus infection); (iv) another chronic disease such as cancer, coronary heart disease, or uncontrolled diabetes; (v) a severe mood disorder; (vi) been pregnant or breastfeeding in the preceding 12 months; or (vii) were morbidly obese (BMI ≥ 40). Severely ill patients with mobility restrictions were visited at home by a research nurse while healthy subjects were invited to a recruiting center for clinical assessment and blood sampling. SOPs were followed for participant identification and invitation, clinical assessments, evaluation of eligibility, phlebotomy, sample transportation and receipt, laboratory blood tests, data entry, processing, storage and sample release^34,35^.

All samples were provided by the UK ME Biobank together with complete health survey SF-36^36^, General Health Questionnaire (GHQ)^37^ and Likert test scores^38^ plus over 30 blood analytical variables. Blood analytics performed at the UK Biobank from aliquots taken at banking blood collection time points, allow matched correlations with parameters evaluated in downstream analysis.

### RNA isolation & RNA quality control

PBMC aliquots were thawed in a 37 °C bath for 1 minute and immediately centrifuged at 300 × g for 5 min. Total RNA was isolated from PBMCs pellets (>10^6^ cells) or isolated EVs (500 µls) using RNAzol (Molecular Research Center) according to the manufacturer’s instructions. RNA yields and quality were assessed using Agilent TapeStation 4200 (Agilent Technologies). PBMCs RNA samples with an RNA Integrity number (RIN) above 7 were included in downstream analysis.

Small RNA distributions were assessed using small RNA kits and the Agilent 2100 bioanalyzer (Agilent Technologies), following manufacturer recommendations. Agilent 2100 bioanalyzer analysis were run at the Centro de Investigación Príncipe Felipe (CIPF) Genomic services in Valencia (Spain).

### EV purification

EVs were isolated from 0.5 ml aliquots of human plasma supernatants (undergoing a single freeze/thaw cycle), upon being centrifuged at 10,000 × g for 10 mins, with Total Exosome Isolation Reagent (TEIR) (Invitrogen by Life Technologies, Cat. 4484450), following manufacturer’s protocol. The process was run in parallel including a proteinase K pretreatment or skipping it for each thawed aliquot.

### EV characterization

Yields and size distribution of isolated EVs were determined using Nanoparticle Tracking Analysis (NTA), light scattering, technology with a Nanosight NS300 instrument (Malvern Panalytical) at the Centro de Investigación Príncipe Felipe (CIPF) Genomic services in Valencia (Spain). Zeta potentials were measured at the same facility with a DLS (Dynamic Light Scattering) Zetasizer Nano ZS (Malvern Panalytical), using a laser wavelength of 532 nm. Capillaries used were cat. Num. DTS 1070 (Malvern Panalytical). EV samples were diluted 1/50 (V/V) in 1X PBS buffer prior to measurements. Zeta potentials were obtained at RT and in triplicate for each analyzed sample. The obtained 90 s videos were recorded in a level 10 camera at the setting of 25 images/s. Video analysis were performed with the NTA version 3.1 and a detection limit of 20.

TEM (transmission electronic microscopy) imaging of EVs was obtained from 1% glutaraldehyde fixed vesicles. A 20 µl drop of each suspension was loaded onto a formvar/carbon-coated grid, negatively stained with 3% (w/v) aqueous phosphotungstic acid for 1 min and observed by transmission electron microscopy (TEM) in a Tecnai Spirit G-2 apparatus; (FEI, Eindhoven, The Netherlands) at the Centro de Investigación Príncipe Felipe (CIPF) Genomic services in Valencia (Spain).

Western-blotting to assess exosomal markers in isolated EVs was performed using the classic Laemmli method with some modifications. In brief, from EVs eluted in 200 µl of 1X PBS from 500 µl of human plasma, 15 µl were lysed in 5X SDS loading buffer and directly loaded into the gel after heating at 95 °C for 5 mins. Primary antibodies used were: anti-CD9 antibody (EX201, Cell Guidance Systems) (1 mg/ml), anti-CD81 antibody (sc-23962, Santa Cruz Biotechnology) (0.2 mg/ml), anti-HSP70 antibody (sc-7298, Santa Cruz Biotechnology) (0.2 mg/ml) and anti-β-Actin antibody (sc-47778, Santa Cruz Biotechnology) (0.1 mg/ml), all these antibodies were used a final dilution of 1/200. As secondary antibodies we used goat anti-mouse IgG-HRP (sc-2005, Santa Cruz Biotechnology) (0.4 mg/ml) using a final dilution of 1/5000.

### Nanostring miRNA profiling & analysis

miRNA profiling was performed in an nCounter NanoString platform (Nanostring Technologies, Seattle, WA, USA), and the nCounter Human miRNA Panel v3 (Nanostring Technologies, GXA-MIR3-12) which evaluates about 800 miRNAs. Each sample was analyzed by using 100 ng of total RNA for hybridization (21 h at 65 °C) in addition to probe pairs consisting of a Reporter Probe, which carry the signal on their 5′ end, and a Capture Probes, which carry biotin on their 3′ end. After hybridization, sample cleanup and digital report counts were performed according to manufacturer’s instructions.

Raw counts were analyzed using Nanostring nSolver version 4.0 software. We calculated geometric mean of negative ligation controls plus two standard deviations for all samples. This value served as a threshold. Thresholds were established as 69 counts in PBMCs and 17 counts in EVs. All count values below this threshold were excluded from normalization. After that, PBMC miRNA input levels were normalized to the geometric mean of the top expressed100 miRNAs. EV miRNA input levels were normalized to six spiked-in controls of non-human miRNAs.

Samples with counts above threshold established values in less than 40% of the miRNAs were eliminated. Next, only miRNAs showing above threshold values in at least 75% of samples, in either group (ME/CFS and HCs), were used for the differential expression analysis in the evaluated blood fraction (either PBMCs or EVs). Statistical differences were assessed as described below.

Heatmaps were built using the Heatmapper website (http://www.heatmapper.ca/expression/), The Spearman Range Correlation was applied to establish the distance across measurements and the Centroid link as a grouping method^64^.

### RT-qPCR validation

Reverse-transcription was performed with the miScript II RT kit (Qiagen, cat. 218161) or the High-Capacity cDNA reverse Transcription kit (Applied Biosystems, cat. 4308228), using 1 µg of total RNA according to manufacturer’s guidelines. cDNAs were used for Real time PCR using miScript SYBR Green PCR kit (Qiagen, cat. 218073), or the PowerUP Sybr Green Master Mix (Applied Biosystems, cat. 100029283) and a Lightcycler LC480 instrument (Roche). Standard amplification conditions were used, including a single hotstart polymerase preactivation cycle at 94 °C for 15 min, followed by 45 amplification cycles each one consisting of 3 steps: denaturation at 95 °C for 15 s, annealing at 50–60 °C for 30 s and extension at 70 °C for 30 s. Sequences of forward miRNA specific primers used were hsa-miR-106b-5p (5′-GTG CTG ACA GTG CAG AT-3′) and hsa-miR-549a: (5′-TGA CAA CTA TGG ATG AGC-3′). As a reverse primer the Universal Primer (UP) provided with the kit was used in all amplifications. U6 RNA amplification levels were used for the relative quantification of the miRNAs amplified. For RT-qPCR of mRNAs, the following primers sets were used: *AGO2* (Forward: 5′-TCG CAC TAT CAC GTC CTC TG-3′ and Reverse: 5′-ATG GCT TCC TTC AGC ACT GT-3′); *MECP2* (Forward: 5′-TGG TAA AAG CCG TCC GG-3′ and Reverse: 5′-GCC CTA ACA TCC CAG CTA-3′) and *NTRK1* (Forward: 5′-TTC AAC GCT CTG GAG TCT-3′ and Reverse: 5′-CAT CAC CGT GGC TGA CT-3′). mRNA levels were normalized to GAPDH levels obtained with primers: Forward: 5′-TGA AGG TCG GAG TCA ACG GAT-3′ and Reverse: 5′-TTC TCA GCC TTG ACG GTG CCA-3′.

### Gene enrichment & pathway analysis

For gene enrichment analysis, potential target genes of the DE miRNAs showing statistical significance (p < 0.05) were identified with the miRDIP database^65^ and the Human miRNA-gene interaction (adjacency) matrix setting. This option demands that target genes present target sites for at least 2 of the DE miRNAs in the set^66^. Next, pathway analysis of the target genes was assessed in the Reactome database^67^. Only target pathways with p values < 0.05 were considered potential effectors of the statistically significant DE miRNAs.

### ROC curves, statistical analysis & plotting

Response operating characteristic (ROC) for each of the miRNAs showing significant DE between ME/CFS and HC individuals (p < 0.05) were assessed with the R package pROC version 1.13.0^68^.

Continuous data are expressed as means ± SD, as indicated. Normal data distribution was assessed using the Shapiro-Wilk test, assuming that p-values > 0.05, followed normal distributions. Statistical differences were determined using t-student or Mann-Whitney tests, depending on whether the data followed or not normal distributions, respectively. To establish group differences, two ranges of significance were applied: p < 0.05 for statistical difference and p < 0.1 for trends or tendencies. Analysis were conducted with the R package version 3.4.2 (R Core Team, 2014)^69^.

Plots were drawn using the R ggplot2 environment version 2.2.1^70^ and the GraphPad Prism 5.0 program. The Venn diagram was drawn with Cytoscape version 3.6.0 and the Venn & Euler diagram package, version 1.0.3.

## Supplementary information

Supplementary Information.

Supplementary dataset 1.

Supplementary dataset 2.

Supplementary dataset 3.

Supplementary dataset 4.

Supplementary dataset 5.

Supplementary dataset 6.

## Data Availability

Nanostring datasets generated during and/or analyzed during the current study are available from the NCBI Gene Expression Omnibus (GEO) database^71^, (Accession Number GSE141770).
