# Supplementary material for: Assessing diagnostic value of microRNAs from peripheral blood mononuclear cells and extracellular vesicles in Myalgic Encephalomyelitis/Chronic Fatigue Syndrome
Source: Sci Rep. 2020 Feb 7;10:2064. doi: 10.1038/s41598-020-58506-5 (PMC7005890; doi:10.1038/s41598-020-58506-5)
Supplement: Supplementary file 1 — Supplementary Information. [file 41598_2020_58506_MOESM1_ESM.pdf]

Supplementary Information

Assessing diagnostic value of microRNAs from peripheral blood mononuclear cells and extracellular vesicles in Myalgic Encephalomyelitis/Chronic Fatigue Syndrome

Eloy Almenar-Pérez, Leonor Sarría, Lubov Nathanson and Elisa Oltra*

Supplementary table S1. Analytic variables measured in donor blood samples (either healthy controls (HC) or ME/CFS patients). Variables showing significant differences (p<0.05) between HC and ME/CFS groups, or those showing a tendency (p<0.1) appear bolded.

	HC		ME/CFS			
**Analytic variable**	**Mean**	**+/- SD**	**Mean**	**+/- SD**	**Range**	**p value**
**Creatine phosphokinase (U/L)**	**88.67**	**30.7**	**59.93**	**25.02**	**167 - 190**	**0.006***
**Alkaline Phosphatase (ALP) (U/L)**	**55.4**	**13.03**	**69.33**	**23.22**	**0 - 33**	**0.055**
**Eosinophils (10^9/L)**	**0.28**	**0.21**	**0.17**	**0.16**	**0.10 - 0.46**	**0.074**
**Free T4 (pmol/L)**	**13.75**	**2.48**	**15.15**	**2.24**	**12.0 - 22.0**	**0.085**
Platelets (10^9/L)	254.73	56.94	239.67	81.23	150 - 400	0.106
Inorganic phosphate (mmol/L)	1.2	0.34	1.05	0.12	0 - 20	0.115
Mean corpuscular hemoglobin concentration (g/L)	322.73	14.41	329.2	10.02	310 - 360	0.128
Potassium (mmol/L)	4.19	0.48	4.03	0.47	3.5 - 5.1	0.175
Rheumatoid factor	19.73	1.03	24.47	17.3	<22	0.179
Monocytes (10^9/L)	0.4	0.12	0.47	0.17	0.1 - 0.8	0.197
Estimated glomerular filtration rate (eGFR)	89.93	6.67	86.33	6.34	>60	0.233
Serum vitamin B12 (pg/mL)	398.67	125.36	544.67	356.46	160 - 680	0.250
Mean corpuscular haemoglobin (pg)	29.04	1.62	29.65	1.31	27.0 - 33.0	0.264
Total calcium (mmol/L)	2.31	0.07	2.43	0.11	2.20 - 2.60	0.301
Albumin (g/L)	43.4	4.19	46.01	2.84	20 - 35	0.326
Haemoglobin (g/L)	127.47	7.03	130.27	8.4	130 - 180	0.330
Basophils (10^9/L)	0.033	0.017	0.04	0.02	0.00 - 0.20	0.331
Adjusted calcium (mmol/L)	2.33	0.08	2.35	0.09	0.93 - 1.45	0.384
Urea (mmol/L)	4.37	0.8	4.02	1.32	2.5 - 7.1	0.413
Creatinine. umol/L.	64.33	9.43	62.27	9.79	49 - 92	0.561
Total bilirubin (umol/L)	9.33	4.78	8.33	4.51	35 - 50	0.601
Neutrophils (10^9/L)	3.53	1.09	3.77	1.48	1.8 - 7.7	0.625
Lymphocytes (10^9/L)	1.82	0.32	1.74	0.58	1.4 - 3.5	0.678
White Blood Cells (10^9/L)	6.06	1.23	6.21	1.88	4.0 - 11.0	0.812
Sodium (mmol/L)	140	2.24	140	2.14	133 - 145	0.818
Mean corpuscular volume (fl)	89.95	4.45	90.07	2.62	80.0 - 98.0	0.929
Free T3 (pmol/L)	4.19	0.53	4.17	0.49	3.9 - 6.5	0.944
Haematocrit	0.4	0.02	0.39	0.02	0.400 - 0.470	0.961
RBC (10^12/L)	4.4	0.28	4.4	0.29	4.40 - 5.40	0.975
C Reactive protein (CRP)	1.07	1.7	0.8	1.08	<9	1.000

Supplementary table S2. RNA yields and integrity from PBMCs. as determined by the 2100 Agilent capillary electrophoresis system (**A**). PBMCs 1-15 corresponded to ME/CFS samples and 16-30 to HCs. RIN <7 are indicated by asterisks. RNA yields from EVs, as determined by the 2100 Agilent capillary electrophoresis system (**B**). EVs 1-15 corresponded to ME/CFS samples and 16-30 to HCs.

A

RNA_ID	conc ng/µl	RIN
PBMC1	70.2	8.5
PBMC2	97.1	7.9
PBMC3	59.8	8.3
PBMC4	60	8.2
PBMC5	57.4	8.4
PBMC6	56	7.4
PBMC7	113	8.3
PBMC8	156	8.2
PBMC9	67.8	7.8
PBMC10	76.3	8.6
PBMC11	64.5	8.4
PBMC12	87.3	8.4
PBMC13	54.9	7.8
PBMC14	64.9	7.7
PBMC15	91.3	7.9
PBMC16	106	8.1
PBMC17	54.5	7.6
PBMC18	82.9	7.9
PBMC19	90.1	8.6
PBMC20	103	8.5
PBMC21	14.3	4.6*
PBMC22	79.4	8
PBMC23	54.5	7.3
PBMC24	80.3	8.7
PBMC25	10.1	4.3*
PBMC26	185	8.3
PBMC27	91	8.3
PBMC28	67.6	8.4
PBMC29	81.6	8.6
PBMC30	75.7	8.3

B

RNA_ID	conc ng/µl	RIN
EXO1	9.44	NA
EXO2	9.17	NA
EXO3	13.2	NA
EXO4	9.01	NA
EXO5	8.46	NA
EXO6	5.63	NA
EXO7	5.46	NA
EXO8	5.35	NA
EXO9	12	NA
EXO10	14.1	NA
EXO11	10.5	NA
EXO12	10.9	NA
EXO13	9.47	NA
EXO14	6.92	NA
EXO15	5.43	NA
EXO16	7.02	NA
EXO17	15.1	NA
EXO18	17.6	NA
EXO19	19.3	NA
EXO20	15	NA
EXO21	13.2	NA
EXO22	17	NA
EXO23	7.44	NA
EXO24	5.09	NA
EXO25	5.19	NA
EXO26	14.5	NA
EXO27	25.7	NA
EXO28	21.2	NA
EXO29	19.3	NA
EXO30	17.4	NA

Supplementary table S3. Differentially expressed miRNAs in PBMCs of ME/CFS and HC PBMCs as determined by nanostring analysis after applying Chauvenet criterion for outliers ($p<0.05$). MiRNAs differentially expressed, both, in PBMCs and EVs are bolded. Those showing a tendency ($p<0.1$) are marked with an asterisk.

Probeset ID	HC (N)	ME/CFS (N)	Fold-change	p-value
**hsa-miR-374a-5p**	435.6 (11)	648.7 (11)	1.49	0.002
Hsa-miR-4516	78.21 (10)	129.3 (13)	1.65	0.003
hsa-miR-644a	209 (12)	135 (12)	-1.55	0.012
hsa-miR-340-5p	151.9 (11)	189.8 (12)	1.25	0.013
hsa-miR-451a	1067 (11)	471.4 (12)	-2.26	0.019
hsa-miR-140-5p	202.45 (12)	263.78 (13)	1.3	0.020
**hsa-miR-4454 & hsa-miR-7975**	8644 (10)	7082 (12)	-1.22	0.021
hsa-miR-18a-5p	69 (11)	82.52 (12)	1.2	0.021
hsa-miR-146a-5p	555.8 (12)	706.7 (12)	1.27	0.024
hsa-miR-549a	201.59 (12)	122.06 (13)	-1.65	0.030
hsa-miR-106a-5p hsa-miR-17-5p	461.1 (12)	516.5 (12)	1.12	0.033
hsa-miR-106b-5p	184.9 (11)	220.6 (12)	1.19	0.037
hsa-miR-361-3p	190.29 (12)	148.57 (13)	-1.28	0.040
hsa-miR-1253	173.2 (12)	113.2 (12)	-1.53	0.045
hsa-miR-590-5p	151.2 (12)	133.8 (12)	-1.13	0.052
**hsa-miR-21-5p***	588.99 (12)	791(13)	1.34	0.060

Supplementary table S4. Differentially expressed miRNAs in ME/CFS and HC in EVs ($p<0.05$) as determined by nanostring analysis, after applying Chauvenet criterion for outliers. MiRNAs differentially expressed, both, in PBMCs and EVs are bolded. Those showing a tendency ($p<0.1$) are marked with an asterisk.

Probeset ID	HC (N)	ME/CFS (N)	Fold-change	p-value
**hsa-miR-4454&miR-7975**	191.7 (12)	465.8 (12)	2.43	0.0043
hsa-miR-150-5p	17.00 (13)	19.86 (12)	1.17	0.0053
hsa-miR-15a-5p	17.00 (13)	30.94 (12)	1.82	0.0212
hsa-miR-183-5p	26.94 (13)	18.98 (11)	-1.42	0.0254
hsa-miR-33a-5p	302.0 (14)	258.2 (12)	-1.17	0.0328
hsa-let-7d-5p	29.14 (12)	50.72 (12)	1.74	0.0329
hsa-miR-423-5p	25.65 (13)	55.02 (12)	2.15	0.035
**hsa-miR-374a-5p**	24.04 (13)	34.85 (12)	1.45	0.0356
hsa-miR-130a-3p	17.06 (12)	32.29 (12)	1.89	0.0408
**hsa-miR-21-5p***	17.23 (13)	28.40 (12)	1.65	0.0547
hsa-miR-320e*	19.16 (13)	26.78 (12)	1.40	0.0578
hsa-miR-203a-5p*	28.70 (14)	21.36 (12)	-1.34	0.0596
hsa-miR-185-5p*	21.74 (13)	33.12 (12)	1.52	0.076
hsa-miR-607*	29.35 (12)	25.32 (11)	-1.16	0.0794
hsa-let-7g-5p*	40.61(13)	70.38 (12)	1.73	0.0864
hsa-miR-126-3p*	73.38 (13)	183.1 (12)	2.50	0.0866
hsa-miR-223-3p*	193.6 (13)	515.9 (12)	2.66	0.0866
hsa-miR-369-3p*	24.93 (13)	20.57 (12)	-1.21	0.0971
hsa-miR-93-5p*	30.63 (13)	47.37 (12)	1.55	0.1048

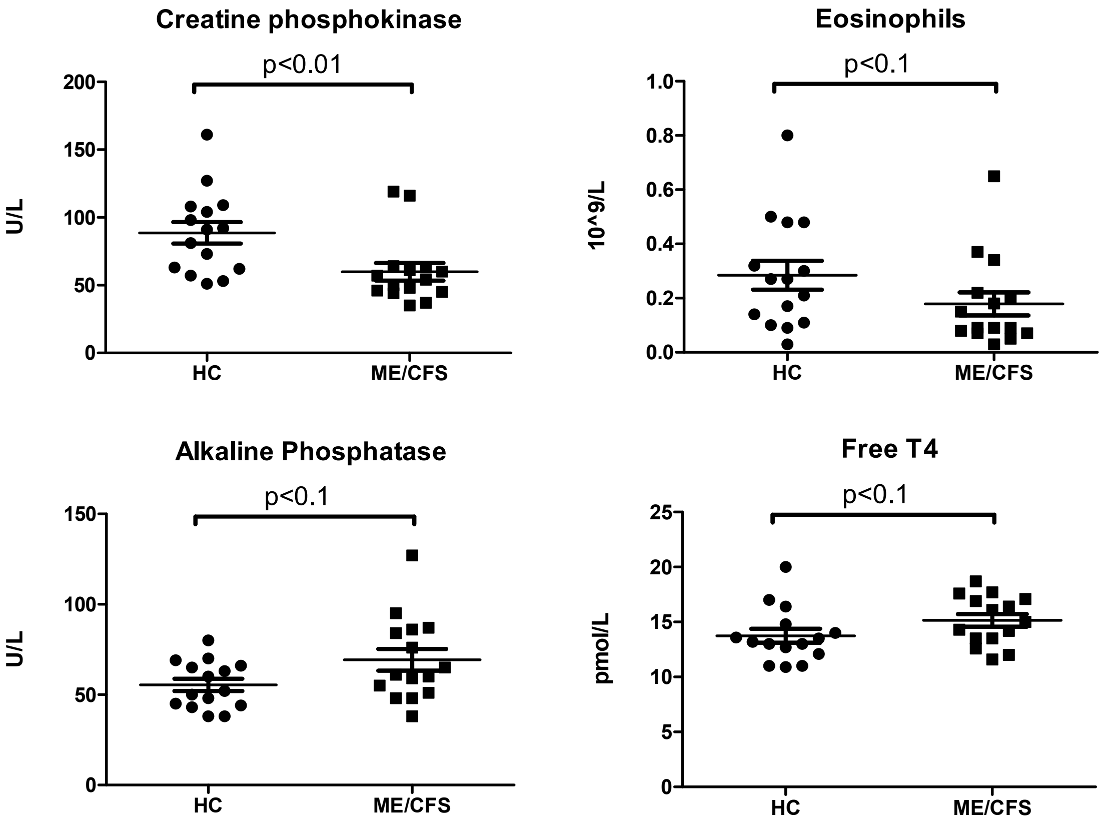

Supplementary figure S1. Clinical parameters showing significant differences ($p<0.05$) or tendencies ($p<0.1$) between groups (HC and ME/CFS, as indicated). Free T4 corresponds to free thyroxine. Means are indicated by horizontal bars, whiskers show standard deviation values (±SD) (t-test).

2100 Agilent expert small RNA

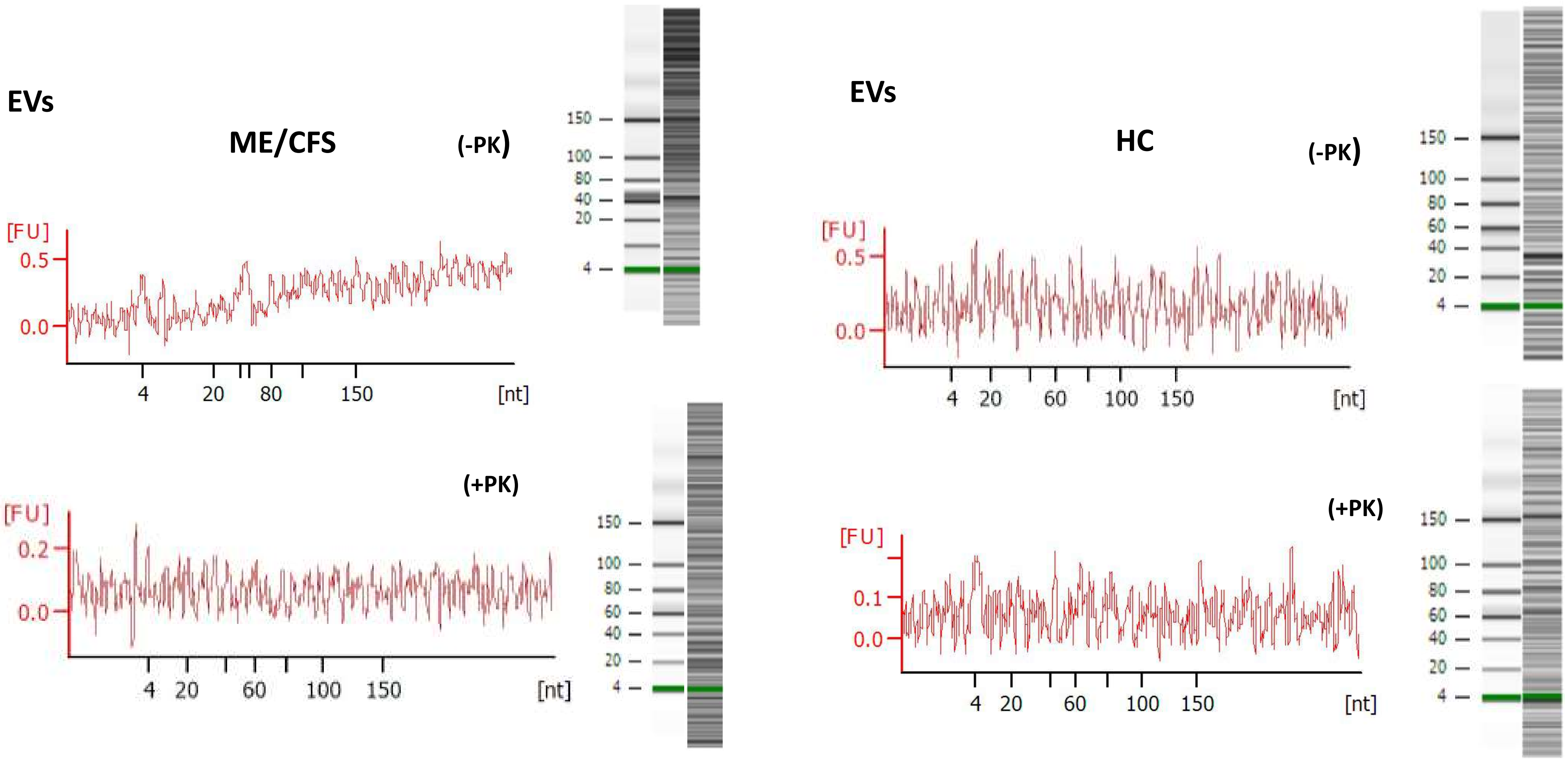

Supplementary figure S2. Representative electropherograms of total RNA extracted from plasma EVs (skipping proteinase K pretreatment (-PK) or including it (+PK), obtained with 2100 Agilent expert small RNA from an ME/CFS patient (left) and a HC (right), as indicated.

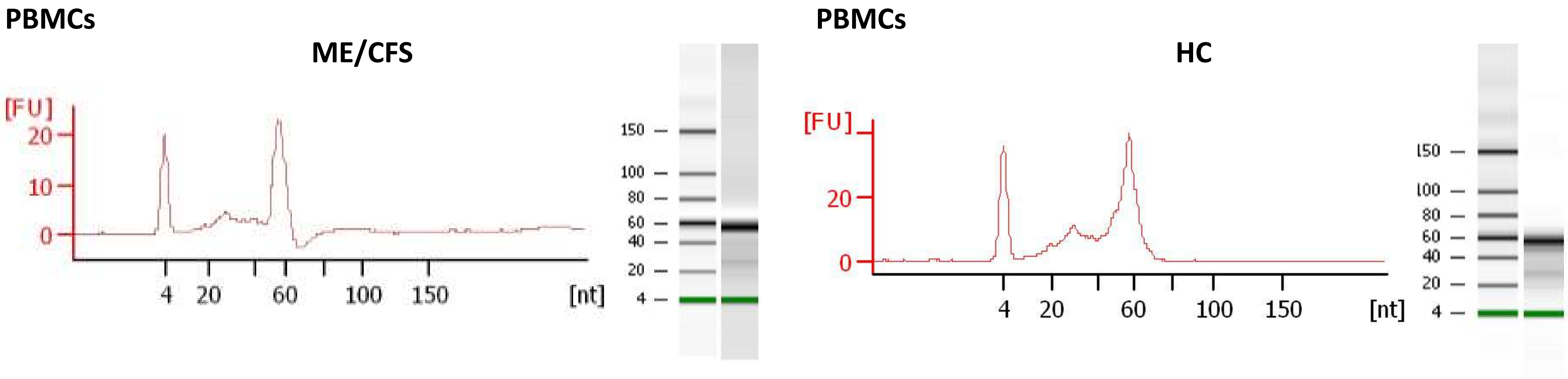

Supplementary figure S3. Representative electropherograms of total RNA extracted from PBMCs obtained with 2100 Agilent expert small RNA from an ME/CFS patient (left) and a HC (right), as indicated.

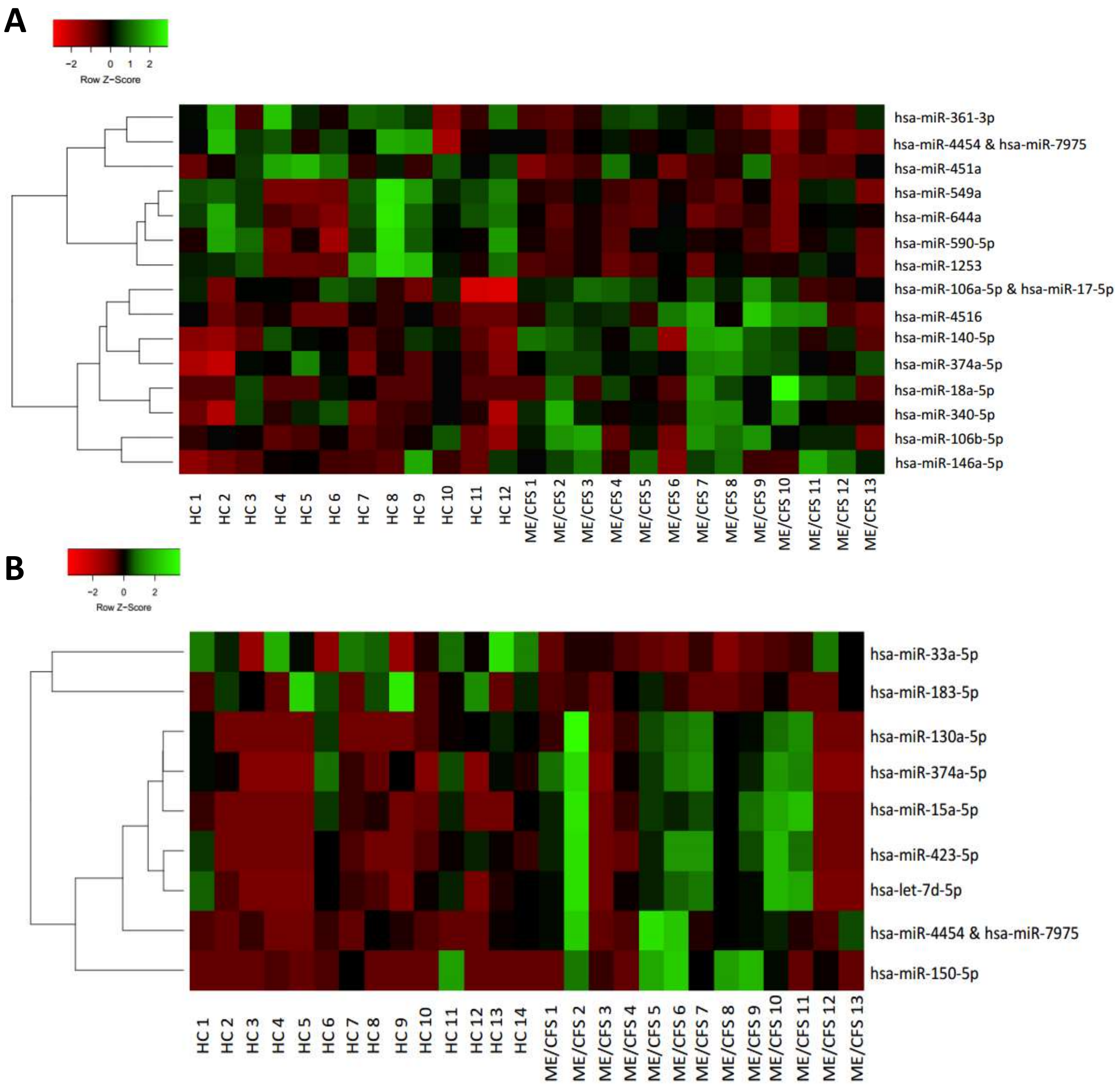

Supplementary figure S4. Heatmaps of significant differentially expressed miRNAs ($p<0.05$) in ME/CFS are shown. Seventeen miRNAs were found in PBMCs (9 overexpressed and 8 under expressed) (**A**); and ten in EVs (8 overexpressed and 2 under expressed) (**B**).
